# Supplementary material for: FERN – a Java framework for stochastic simulation and evaluation of reaction networks
Source: BMC Bioinformatics. 2008 Aug 29;9:356. doi: 10.1186/1471-2105-9-356 (PMC2553347; doi:10.1186/1471-2105-9-356)
Supplement: Additional file 1 — FERN distribution, Version 1.3. This archive contains the FERN source code and binaries as well as documentation and example models in FernML and SBML. [file 1471-2105-9-356-S1.zip › fern/doc/javadoc/fern/simulation/observer/package-tree.html]

fern.simulation.observer Class Hierarchy


---


|  |  |  |
| --- | --- | --- |
| **Overview** \| **Package** \| Class \| Use \| **Tree** \| **Deprecated** \| **Index** \| **Help** | |  |
| **PREV**   **NEXT** | **FRAMES**    **NO FRAMES**     **All Classes** |  |


---


## Hierarchy For Package fern.simulation.observer

**Package Hierarchies:**: All Packages

---

## Class Hierarchy

- java.lang.**Object**
  - fern.simulation.observer.**Observer**
    - fern.simulation.observer.**AmountAtMomentObserver** (implements fern.simulation.observer.GnuPlotObserver, fern.simulation.controller.SimulationController)- fern.simulation.observer.**FireTypeObserver** (implements fern.simulation.observer.GnuPlotObserver)- fern.simulation.observer.**InstantOutputObserver**- fern.simulation.observer.**IntervalObserver** (implements fern.simulation.observer.GnuPlotObserver)
            - fern.simulation.observer.**AmountIntervalObserver**- fern.simulation.observer.**MultiAmountIntervalObserver**- fern.simulation.observer.**ReactionIntervalObserver**
            - fern.simulation.observer.**LeapObserver** (implements fern.simulation.observer.GnuPlotObserver)- fern.simulation.observer.**RandomNumberGeneratorCallObserver** (implements fern.simulation.observer.GnuPlotObserver)- fern.simulation.observer.**TriggerObserver**

## Interface Hierarchy

- fern.simulation.observer.**GnuPlotObserver**

---


|  |  |  |
| --- | --- | --- |
| **Overview** \| **Package** \| Class \| Use \| **Tree** \| **Deprecated** \| **Index** \| **Help** | |  |
| **PREV**   **NEXT** | **FRAMES**    **NO FRAMES**     **All Classes** |  |


---
